# Chronic periaortitis (retroperitoneal fibrosis) concurrent with giant cell arteritis: a case report

**DOI:** 10.1186/1752-1947-8-167

**Published:** 2014-05-27

**Authors:** Ioannis Protopsaltis, Athanasios Sotiropoulos, Argyrios Foteinos, Kassiani Manoloudaki, Kiriaki Boki, Garifallia Linardaki, Athanasia Papazafiropoulou, Stavros Antonopoulos

**Affiliations:** 1 Department of Internal Medicine, Tzanio General Hospital of Piraeus, Piraeus, Greece; 2 Rheumatology Department, Sismanoglio Hospital, Athens, Greece; 3 3rd Department of Internal Medicine and Center of Diabetes, General Hospital of Nikaia “Ag. Panteleimon”, Nikaia, Greece

**Keywords:** Giant cell arteritis, Periaortitis, Retroperitoneal fibrosis

## Abstract

**Introduction:**

Giant cell arteritis is the most common form of large-vessel vasculitides. However, it is probable that extracranial involvement is underdiagnosed in patients with classical giant cell arteritis. In the recent literature most cases of giant cell arteritis have been described in conjunction with aortic aneurysms or dissections. Nonetheless the coexistence of giant cell arteritis and retroperitoneal fibrosis is extremely rare. Here, we describe a case of giant cell arteritis at a very early clinical stage, in a woman with coexistence of retroperitoneal fibrosis.

**Case presentation:**

We report a case of giant cell arteritis at a very early clinical stage, in a 47-year-old Greek woman with coexistence of retroperitoneal fibrosis who was admitted to our hospital with a history of high-grade fever and mild right periumbilical abdominal pain for the past 30 days. In the context of fever of unknown origin, an abdomen computed tomography was ordered. A temporal artery biopsy was also performed because during hospitalization she complained of a headache. Examination of eosin and hematoxylin slides from biopsy specimens of her temporal artery, showed lesions consisting of predominantly lymphocytes, few plasma cells and occasional polymorphonuclear leucocytes. In addition no giant cells were detected in examining biopsies at multiple levels. This was consistent with giant cell arteritis according to the American college of Rheumatology criteria. An abdomen computed tomography revealed the presence of a retroperitoneal soft-tissue mass located anteriorly to the upper infrarenal aorta at the site of the scintigraphic uptake. The computed tomography and magnetic resonance imaging characteristics of the mass were consistent with retroperitoneal fibrosis, and its morphology suggestive of benignity. Our patient started oral prednisolone and was afebrile from day one.

**Conclusions:**

In our experience this is the first case of retroperitoneal fibrosis due to giant cell arteritis occurring at the same time. Involvement of the aorta (aortitis) and its branches has been also observed in a subset of patients with giant cell arteritis. In addition, giant cell arteritis has been associated with a markedly increased risk of aortic aneurysm particularly thoracic aortic aneurysm.

## Introduction

Giant cell arteritis (GCA) is the most common form of large-vessel vasculitides. Although GCA involves branches of the external carotid artery, involvement of the aorta has also been observed. It is therefore probable that extracranial involvement is underdiagnosed in patients with classical GCA. In the recent literature most cases of GCA have been described in conjunction with aortic aneurysms or dissections. Nonetheless the coexistence of GCA and retroperitoneal fibrosis (RPF) is extremely rare. Here, we describe a case of GCA at a very early clinical stage, in a woman with coexistence of RPF.

## Case presentation

A 47-year-old Greek woman was admitted to our hospital with a history of high-grade fever and mild right periumbilical abdominal pain for the past 30 days. Fever spikes were daily up to 41°C, accompanied with chills and malaise. She reported no traveling during the last year, pets or contacts with other animals, diarrhea or vomiting. She also denied any cough or dyspnea, photophobia, headaches or musculoskeletal pains.

On physical examination she was fully alert. Her vitals were a blood pressure of 110/80mmHg, oxygen saturation of 98%, temperature of 39.5°C, and heart rate of 120 beats per minute. Her abdomen was soft, with no signs of peritoneal inflammation. Both her temporal arteries were prominent, with strong pulses and no nodularity.

On admission her chest X-ray and electrocardiogram were normal. The laboratory results were as follows: white blood cells count 9000/mm^3^, hemoglobin 10.2g/dL, platelets 280,000/μL, erythrocyte sedimentation rate 75mm/hour, C-reactive protein (CRP) 82.2mg/dL. Serum levels of electrolytes, albumins, total bilirubin, and renal and liver function tests were normal. All cultures were sterile. The results of tuberculin skin tests, virology and bacterial markers as well as immunoassays were negative. Serum protein electrophoresis was compatible with an acute inflammatory reaction. Her fever was unresponsive to broad-spectrum intravenous antibiotics.

In the context of fever of unknown origin, an abdomen computed tomography (CT) was ordered. A temporal artery biopsy was also performed because during hospitalization she complained of a headache.

**Figure 1:**
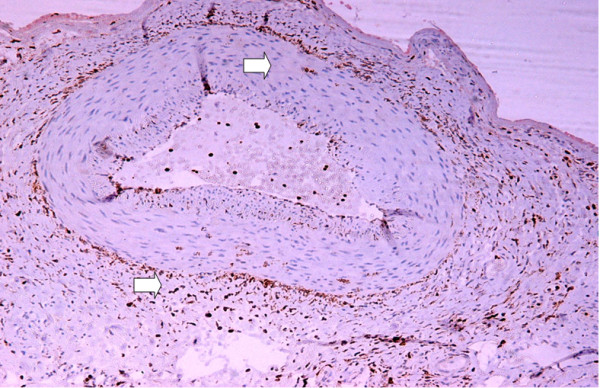
CD68(+) macrophages within the wall of the temporal artery (arrows).

Examination of eosin and hematoxylin slides from the biopsy specimens of the temporal artery, showed thrombus formation in the lumen, focal intimal thickening and long breaks in the internal elastic lamina, as well as a transmural inflammation consisting of predominantly lymphocytes, few plasma cells and occasional polymorphonuclear leucocytes. In addition no giant cells were detected in examining biopsies at multiple levels. This was consistent with GCA according to the American College of Rheumatology criteria [1]. Despite the fact that the above criteria for establishing the diagnosis of GCA were met, immunohistochemical staining for the macrophage marker CD68 revealed (Figure 1) CD68+ macrophages present within the artery wall, which further confirmed the diagnosis of GCA [2].

**Figure 2:**
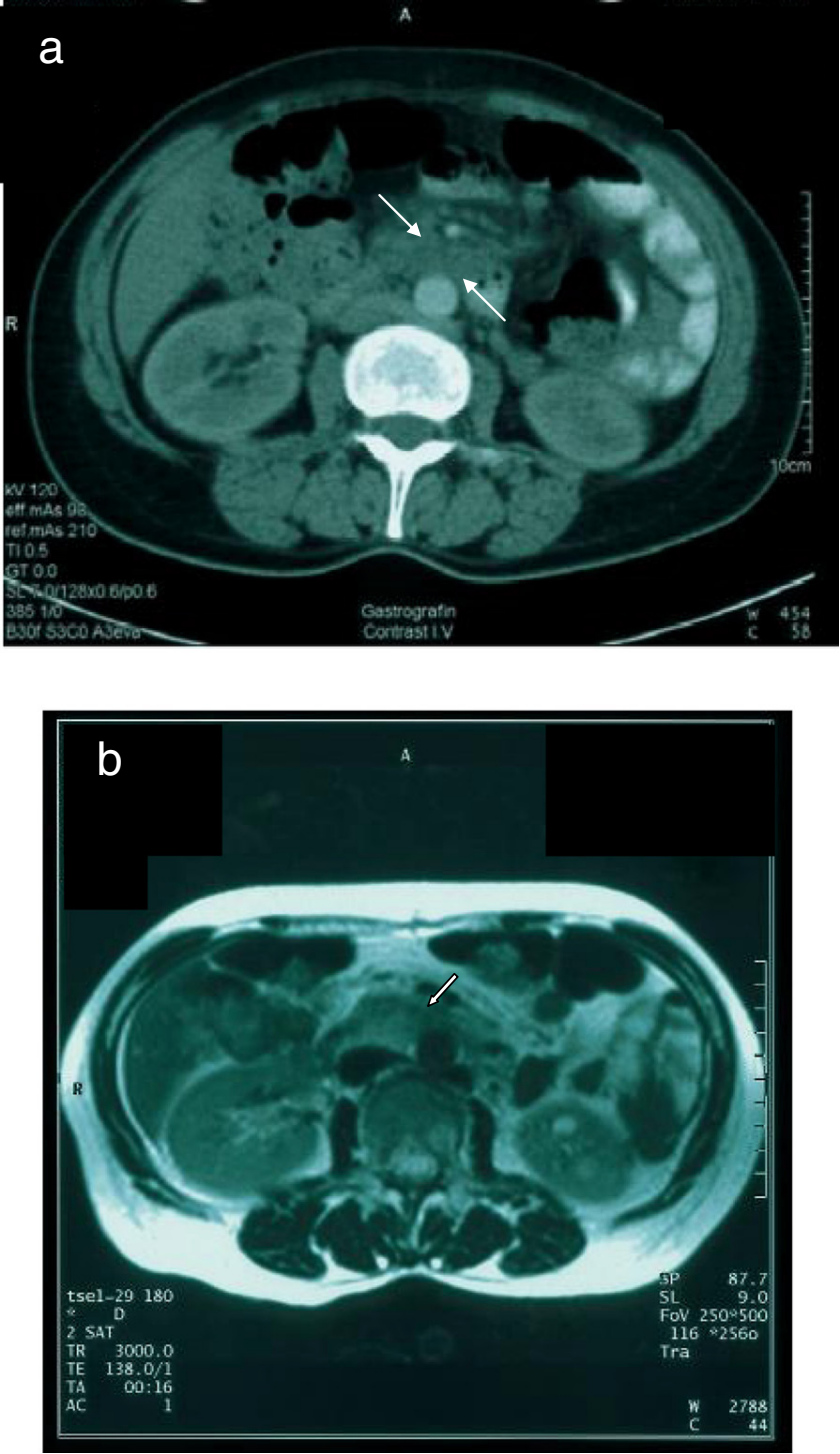
**(a) Computed tomography images show presence of a low-attenuation mass engulfing the anterior surface of the infrarenal aorta (arrows).** Note there is no apparent soft-tissue enhancement of the lesion. **(b)** Axial T2-weighted magnetic resonance image shows low to intermediate signal of the mass (arrow).

The abdomen CT revealed the presence of a retroperitoneal soft-tissue mass located anteriorly to the upper infrarenal aorta at the site of the scintigraphic uptake. The lesion had well-demarcated irregular margins and nearly engulfed the anterior surface of the aorta. After intravenous contrast administration of intravenous contrast agent, no enhancement of the mass was evident (Figure 2a). An abdomen magnetic resonance imaging (MRI) was also performed: MRI signal characteristics of the lesion were similar to those of a fibrotic process. More specifically, the lesion had diffusely low signal on T1-weighted images and low to intermediate signal on T2-weighted images. After intravenous administration of gadolinium, lack of soft-tissue enhancement was also confirmed (Figure 2b). The CT and MRI characteristics of the mass were consistent with RPF, and its morphology suggestive of benignity [3].

## Discussion

According to the above findings, a diagnosis of coexistence of GCA at a very early clinical stage and RPF was set. The patient when started on 60mg oral prednisolone was afebrile from day one; whereas her abdominal pain and headache disappeared completely. Her CRP levels returned to baseline, her hemoglobin normalized and she was discharged for further consultation with a rheumatologist.

GCA is an inflammatory vasculitis seen predominantly in the elderly that affects preferentially medium and large arteries. Our patient, aged 47 years, is at the younger end of the GCA spectrum. GCA commonly involves branches of the external carotid artery; this is why headache, as well as scalp tenderness, jaw claudication and facial pain, occurs in 50% to 75% of patients with GCA.

Involvement of the aorta (aortitis) and its branches has been also observed in a subset of patients with GCA [4]. Aortitis is the term used to define inflammation of one or more layers of the aortic wall. Therefore, it has been suggested that there are two variants of GCA: the typical pattern responsible for temporal arteritis and an atypical variant involving the aorta [5]. In addition, GCA has been associated with a markedly increased risk of aortic aneurysm particularly thoracic aortic aneurysm [5].

A second major category of aortic inflammatory disease is chronic periaortitis that has a predilection for the abdominal aorta. The causes of chronic periaortitis have generally been considered distinct from the entities that cause thoracic aortitis. In addition, periaortitis has several clinical presentations such as idiopathic RPF, inflammatory abdominal aortic aneurysm, and perianeurysmal RPF [6]. Hence the term periaortitis is also used as synonymous for RPF [6].

In our experience, this is the first case of RPF due to GCA occurring at the same time. In addition, in our case, the two clinical entities appear not only to evolve simultaneously, but a simultaneous remission was observed, after corticosteroid treatment. Indeed the patient’s abdomen MRI on 1-year follow-up revealed absence of enhancement and the relatively low T2 signal of the lesion reflecting a lower degree of associated active inflammation, a feature indicating a favorable therapeutic response [3].

Chronic periaortitis is characterized by inflammatory involvement of the outer layer of the aorta and surrounding tissues. On histopathological examination there is a marked aortic adventitial inflammation with inflammatory involvement of vasa vasorum, involving also retroperitoneal small vessels. However, pathologic similarities have been found between chronic periaortitis and large-vessel vasculitis, regarding vessel wall inflammation and involvement of both medial and adventitial layers; whereas vasa vasorum vasculitis may have a key role in the initiation of the immune response, resulting in pathogenesis of aortic involvement in GCA [7]. Αdventitial inflammation and involvement of the vasa vasorum, are common findings encountered in both clinical entities, highlighting even more the autoimmune character of chronic periaortitis [8].

Furthermore, there is growing evidence that chronic periaortitis may be the manifestation of a systemic disease rather than a localized reaction. In particular the presence of antibodies in chronic periaortitis (antinuclear antibodies, antineutrophil cytoplasmic and antismooth muscle antibodies) and its frequent association with other autoimmune diseases [8] further supports the concept of an autoimmune process participating in the pathogenesis of chronic periaortitis. This is also suggested by the reported association with HLA-DRB*03 [9] and the corticosteroid-responsive nature of the disease. Even though initially the pathogenesis of chronic periaortitis had been attributed to a local inflammatory reaction to antigens, such as ceroid and oxidized low-density lipoproteins (LDL) located within the arteriosclerotic plaques, findings reported by other researchers do not support this notion [10]. Chronic periaortitis can affect patients without atherosclerosis, such as pediatric patients, whereas a non-statistically significant difference was found in anti-oxidized-LDL antibody levels between patients with chronic periaortitis and controls [10].

In fact, chronic periaortitis has been associated with systemic vasculitides such as Wegener’s granulomatosis and polyarteritis nodosa, immunoglobulin (Ig)G4-related systemic disease, lupus erythematosus and antiphospholipid syndrome [11]. In our patient, measurement of serum IgG4 was not performed. As previously reported serum immunoelectrophoresis IgG levels were normal. Only in the case of polyclonal hypergammaglobulinemia is there a need to determine serum Ig levels and, if available, IgG subclasses [11]. In addition, our patient was not found to have autoimmune pancreatitis, or other lesions related with IgG4 disease [11].

With a histologically proven diagnosis of GCA and a CT-documented retroperitoneal mass indicative of RPF in our patient, we assumed that autoimmunity seems to be the link between these two apparently different clinical entities. Consequently, although periaortitis (RPF) is idiopathic in most cases, in this case it seems to be secondary occurring in the context of a large-vessel vasculitis such as GCA. Indeed, some cases of RPF are today considered to be secondary to systemic vasculitides caused by autoimmune-mediated mechanisms [12]. Moreover, recently Tölle *et al*. reported a case of patient with GCA followed by idiopathic RPF approximately 2 years later [13].

Currently, no evidence is available about the usefulness of positron emission tomography (PET) or MRI in monitoring patients with GCA aortitis at regular intervals after the discontinuation of treatment in order to prevent long-term complications [14]. By contrast, aortitis has been found to be a feature in 23% to 65% of GCA patients [15]. Since the previous reports have shown frequent aortic involvement even in the early stages of the disease, it is reasonable that all GCA patients should have an initial diagnostic workup focused on the presence of aortitis, given that GCA aortitis remains largely underestimated. Modern imaging modalities such as PET, MRI or contrast-enhanced CT of the abdomen or chest should be carried for establishing the early diagnosis of GCA aortitis and other aortic abnormalities [15]. In addition, it should be kept in mind that the presentation with classic cranial symptoms and signs of temporal arteritis has been proven to be a negative predictor of an aortic complication.

## Conclusions

The coexistence of GCA and RPF is rare but this does not indicate that it should not be in the differential diagnosis of fever of unknown origin. However, data from more cases need to be collected in order to clarify the actual prevalence of RPF among patients with GCA, the response to treatment and the prognosis of these patients.

## Consent

Written informed consent was obtained from the patient for publication of this case report and any accompanying figures. A copy of the written consent is available for review by the Editor-in-Chief of this journal.

## Abbreviations

CRP: C-reactive protein; CT: Computed tomography; GCA: Giant cell arteritis; Ig: Immunoglobulin; LDL: Low-density lipoproteins; MRI: Magnetic resonance imaging; PET: Positron emission tomography; RPF: Retroperitoneal fibrosis.

## Competing interests

The authors declare that they have no competing interests.

## Authors’ contributions

IP and AP were involved in the initial writing of the manuscript. SA provided major editing changes. AS, AF, KM, GL and KB were primarily involved in the care of our patient. All authors have read and approved the final version of the manuscript.
